# Feasibility of an exercise program in endocrine-treated metastatic breast cancer patients with overweight: protocol for the FEMA study

**DOI:** 10.1186/s40814-025-01621-9

**Published:** 2025-04-02

**Authors:** May Wissing, Pernille Skovlund, Susanne Drysdale, Ali Amidi, Robert Zachariae, Tinne Laurberg, Signe Borgquist

**Affiliations:** 1 Department of Oncology, Aarhus University Hospital, Palle Juul-Jensens Boulevard 99, Aarhus N, Denmark; 2 Department of Clinical Medicine, Aarhus University, Palle Juul-Jensens Boulevard 11, Aarhus N, Denmark; 3 Unit for Psychooncology and Health Psychology (Epos), Department of Oncology, Aarhus University Hospital, Palle Juul-Jensens, Boulevard 99, Aarhus N, Denmark; 4 Department of Psychology and Behavioural Science, Aarhus University, Bartholins Allé 11, Aarhus C, Denmark; 5 Steno Diabetes Center Aarhus, Palle Juul-Jensens Boulevard 11, Aarhus N, Denmark; 6 Department of Pathology, Aarhus University Hospital, Palle Juul-Jensens, Boulevard 35, Aarhus N, Denmark

**Keywords:** Metastatic breast cancer, Endocrine treatment, Overweight, Obesity, Exercise, Physical activity, Intervention

## Abstract

**Background:**

Many patients with metastatic breast cancer can live relatively long lives but are challenged by treatment- and cancer-related side effects such as weight gain, physical deconditioning, and reduced quality of life, possibly affecting survival. In particular, endocrine treatments are associated with an increased risk of weight gain and adverse metabolic effects. There is a need for interventions to prevent side effects among patients with disseminated breast cancer. Exercise is found to be effective in improving quality of life, metabolic health, and body composition in the curative setting, yet evidence in the metastatic setting is sparse. The aim of this study is to assess feasibility of a 12-week exercise intervention for metastatic breast cancer patients with overweight receiving endocrine therapy and to explore potential effects on metabolic health, body composition, physical performance, obesity-related biomarkers, and patient-reported outcomes.

**Methods:**

The FEMA study is a randomized controlled feasibility trial in which 21 endocrine-treated patients with metastatic breast cancer and overweight will be randomly assigned in a 2:1 ratio to either a 12-week training program with three weekly training sessions (intervention), or usual care (control), which includes standard clinical follow-up and supportive care without structured exercise. Feasibility will be assessed based on recruitment rate, adherence, retention, and acceptability, employing both quantitative and qualitative approaches for data collection. Participants’ experiences will be explored by interviews and analyzed based on content analysis. Data are collected from blood samples, bioelectrical impedance analysis, physical performance tests, blood pressure measurements, and validated questionnaires on health-related quality of life, self-efficacy for coping with cancer, and sleep quality for explorative analyses.

**Discussion:**

The planned study will allow us to determine whether this 12-week exercise intervention is feasible in endocrine-treated metastatic breast cancer patients with overweight and explore potential effects on metabolic health, body composition, physical performance, obesity-related biomarkers, and patient-reported outcomes. Information from feasibility outcomes will inform the design of a future definitive randomized controlled trial.

**Trial registration:**

Retrospectively registered on March 6, 2024, at ClinicalTrials.gov (NCT06343987).

**Supplementary Information:**

The online version contains supplementary material available at 10.1186/s40814-025-01621-9.

## Background

A substantial increase in the prevalence of overweight and obesity globally is also reflected in Scandinavia, including Denmark [1]. According to the Danish Health Authority, 53% of the Danish adult population have a body-mass index (BMI) ≥ 25 kg/m^2^ and are, by definition, overweight, whereas 18.5% have a BMI ≥ 30 kg/m^2^ and are considered to have obesity [2]. Overweight presents a significant individual and public health challenge, as excess weight is associated with an increased risk of many health issues, including type 2 diabetes, cardiovascular diseases, and several types of cancers [3–6]. In the case of breast cancer (BC), which is the most common cancer in women worldwide [7], accounting for approximately 2.3 million new cases in 2022 [8], overweight is not only associated with an increased risk of developing BC but also with a less favorable prognosis [9–11]. At diagnosis, women with overweight are more likely to have more advanced BC [12] and face an elevated risk of developing distant metastases compared to BC patients with normal weight [9].

Several factors may explain the inferior prognosis observed among BC patients with overweight. Women who are overweight tend to have higher insulin levels, systemic low-grade inflammation, and, in postmenopausal women, higher circulating levels of estrogen [13, 14]. Hyperinsulinemia and chronic low-grade inflammation possibly contribute to more aggressive BC and promote tumor progression [15, 16]. Additionally, systemic cancer treatments, e.g., chemotherapy and endocrine-based therapy, may be less effective in BC patients with overweight [17, 18], altogether increasing the risk of the cancer metastasizing.

For women with metastatic breast cancer (MBC), improved cancer care is a high priority [19]. Despite having an incurable disease, many patients can live relatively long lives after diagnosis, with a median overall survival of 57 months for patients with estrogen receptor-positive (ER +) MBC [20]. However, patients with MBC often experience significant cancer- and treatment-related side effects, such as metabolic disruption and weight gain, possibly related to endocrine treatment [21, 22], fatigue, depression, and physical deconditioning, adversely affecting their health-related quality of life (HRQoL) [23–26]. Impaired HRQoL may lead to treatment adjustment or discontinuation, which can affect survival negatively [27].

One potential avenue to combat these adverse outcomes in patients with MBC is through exercise. In recent international guidelines, exercise is recommended in the curative setting alongside and after conventional treatments to reduce side effects and improve survival [28, 29]. However, no recommendations are provided in the metastatic setting, as evidence of the beneficial effects of exercise in these patients is scarce. While multiple prospective studies have investigated exercise interventions in the curative BC setting, patients with MBC are often not included, even though exercise is found to be safe [30], including for those with bone metastases [31].

A few studies have investigated the effects of exercise in the MBC setting with inconsistent results. In the PREFERABLE-EFFECT study by Hiensch et al. [32], 357 patients were randomized to a 9-month supervised exercise program versus usual care. While exercise was found to significantly improve HRQoL and fatigue, the authors did not examine outcomes on body composition and metabolic health. Scott et al. [33] randomized 65 patients with MBC in treatment with chemotherapy to a 12-week aerobic training program versus a stretching control group. Aerobic training was found to be safe, however not feasible, as almost one-third (N = 9/33) in the intervention group discontinued due to disease progression, lack of motivation or pain.

In a pilot trial, Sheean et al. [34] investigated a 12-week exercise and nutritional intervention versus a wait-list control condition in patients with MBC (*N* = 40), providing lifestyle coaching and intervention support for home-based training. The intervention was deemed feasible and safe and resulted in improvements in HRQoL, fatigue, endocrine symptoms, and visceral fat mass. Results from a full-scale study have yet to be published.

Ligibel et al. [35] conducted a 16-week randomized study of partially supervised aerobic training versus usual care in 101 patients with MBC. The exercise group showed no significant improvements in physical activity or HRQoL. However, a post hoc analysis revealed that patients on endocrine therapy had better cardiorespiratory fitness outcomes and lower dropout rates. The authors concluded that the study’s heterogeneous population, with women at different treatment stages and therapies, led to variations in physical activity and functional outcomes, reducing the power to detect changes. Therefore, they suggest that future studies should focus on more homogenous groups of MBC patients, particularly those on endocrine therapy.

A subgroup of patients with MBC for whom exercise could be particularly beneficial are those who receive endocrine treatment and have overweight or obesity. As seen in Ligibel et al., exercise may be more feasible for those MBC patients who receive endocrine treatment than those receiving chemotherapy. Furthermore, in this patient population, there is a considerable need to mitigate treatment-, cancer- and obesity-related adverse effects on HRQoL, metabolic health, and body composition and to potentially improve survival. In the curative setting, one study investigated a 16-week combined aerobic and resistance exercise intervention in BC survivors with overweight receiving endocrine therapy (*N* = 100). They found significant improvements in metabolic syndrome, sarcopenic obesity, physical fitness, and HRQoL [36, 37]. This needs to be tested in the advanced setting as well.

In this context, we hypothesize that a supervised exercise program is feasible and can improve metabolic health, body composition, and patient-reported outcomes in patients with MBC and overweight treated with endocrine therapy, ultimately improving cancer care in the advanced setting. Therefore, we aim to (1) assess the feasibility of a 12-week PA program for women with MBC and overweight receiving endocrine-based treatment in terms of recruitment rate, adherence, retention, and acceptability and (2) investigate the potential effects of the exercise program on metabolic health, body composition, physical performance, obesity-related biomarkers and patient-reported outcomes such as HRQoL, cancer-related self-efficacy, and sleep quality.

## Method

**Fig. 1:**
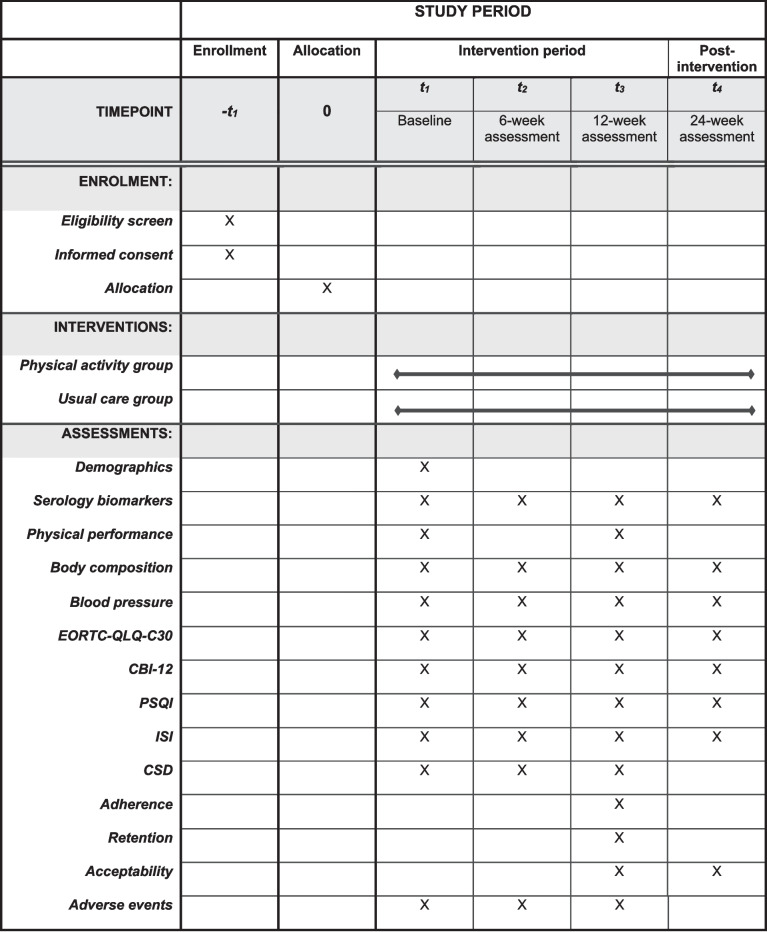
SPIRIT-flow diagram: schedule of enrollment, intervention, and assessment Notes: EORTC-QLQ-C30, The European Organization for Research and Treatment of Cancer QLQ-C30; CBI-12, Cancer Behavior Inventory 12; PSQI, Pittsburg Sleep Quality Index; ISI, Insomnia Severity Index; CSD, Consensus Sleep Diary

The present protocol is based on the Standard Protocol Items: Recommendations for Interventional Trials (SPIRIT) [38, 39] and the Consolidated Standards of Reporting Trials (CONSORT) statement extension to randomized pilot and feasibility trials [40], following the editorial guide from Thabane and Lancaster [41]. A SPIRIT flow diagram is provided in Fig. 1, and the SPIRIT checklist can be found in the additional material (Additional File 1).

### Study design

**Fig. 2:**
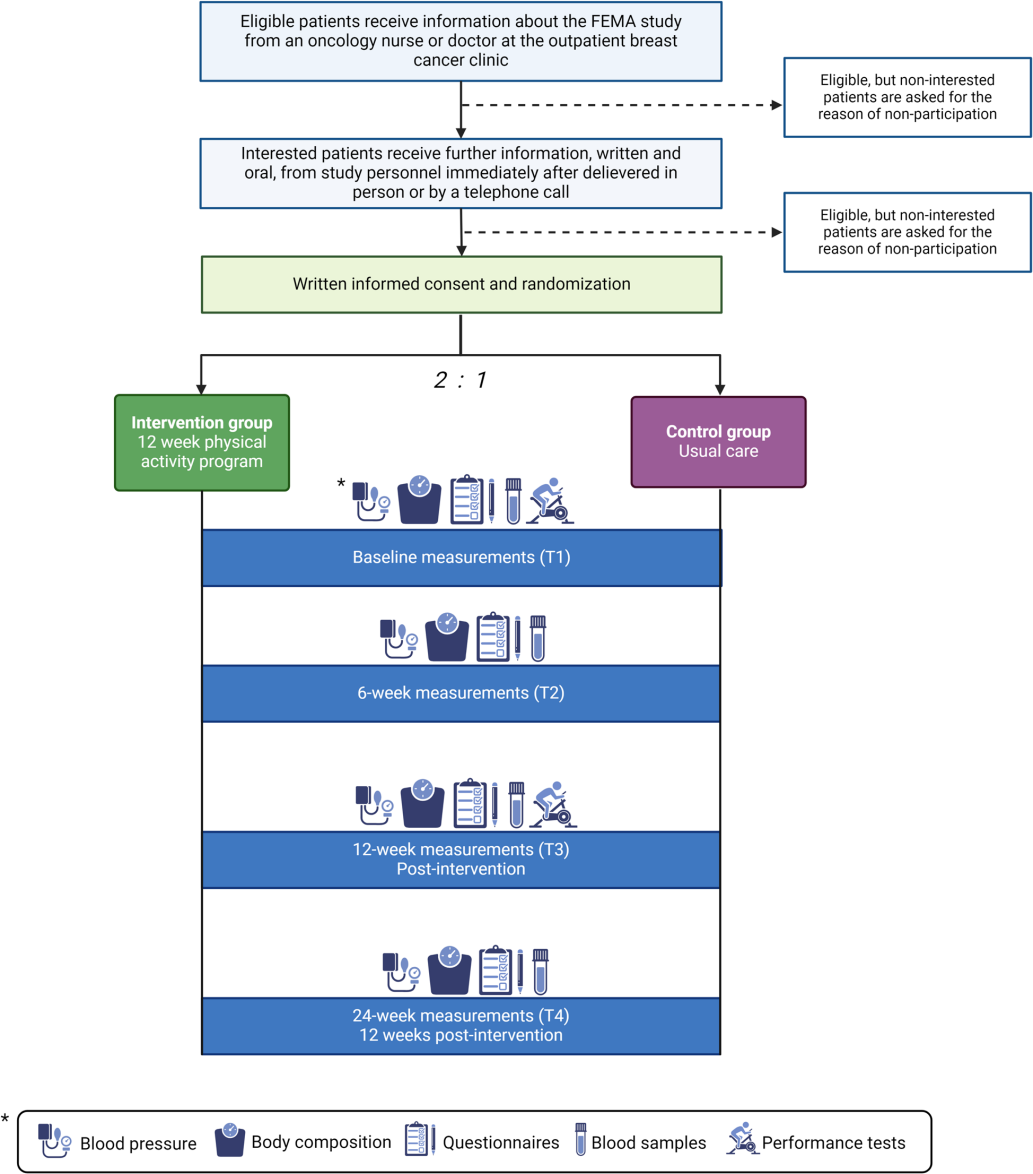
Study design

The FEMA study (Feasibility of Exercise in patients with Metastatic Breast Cancer and Adiposity) is a parallel-group, randomized controlled, feasibility trial with two arms. After providing informed consent, eligible participants are randomly assigned in a 2:1 ratio to the interventional arm, consisting of usual care plus a 12-week exercise program or a usual care-only control arm. Outcome measures are collected at study inclusion (baseline), mid-intervention (6 weeks), post-intervention (12 weeks), and at a 12-week follow-up after the intervention concludes (24 weeks) The study design and timeline are illustrated in Fig. 2 further below.

### Participants and setting

The target population for this study is patients with MBC who are overweight or obese (BMI ≥ 25) and who receive first-line endocrine treatment. We will recruit participants (*N* = 21, see “ Sample size” section below) from the breast cancer outpatient clinic at the Department of Oncology, Aarhus University Hospital, Denmark. The training facilities for the exercise program are provided by the Steno Diabetes Center Aarhus, located at Aarhus University Hospital.

### Eligibility criteria

Patients are eligible for inclusion if they meet the following criteria: (1) diagnosed with MBC; (2) in active first-line endocrine treatment; (3) BMI of 25 kg/m^2^ or higher; and (4) performance status of Eastern Cooperative Oncology Group (ECOG) of 0–1. Participants are excluded according to the following criteria: (1) pregnancy; (2) physical health condition making it either unsafe to participate or preventing them from full participation in the exercise program; and (3) insufficient Danish language skills.

### Recruitment

Project personnel will screen the outpatient BC clinic’s appointment list daily to identify potentially eligible participants. Eligible participants are highlighted in the list to make the attending physician or nurse aware of eligibility. The attending physician or nurse is responsible for informing the patient about the study. If the patient is interested, project personnel will provide detailed information about the study procedures and answer any questions. Eligible participants receive a study information sheet describing the study objectives and methods in detail. The patient is then offered time to consider participation, and a follow-up meeting is scheduled. All participants must provide written consent before participating in any study-related procedures. Those who opt not to participate will be documented in a screening log stored on an internal server. All participants will be compensated for parking or public transportation costs related to study participation.

### Randomization

Recruited participants undergo non-stratified, simple randomization in a 2:1 ratio to either intervention or usual care. The randomization sequence is computer-generated independently through an algorithm used by the Clinical Trial Unit at Aarhus University. When the participants have been randomized, their allocation will be locked in the Research Electronic Data Capture (REDCap) software [42, 43]. The randomization process is not blinded for researchers or participants, as it is not compatible with the study design. Randomization allocation is delivered orally to participants.

### Sample size

As this study is a feasibility trial, no formal sample size calculation was performed, which aligns with published recommendations [40, 41, 44]. The chosen sample size of 21 participants was based on practical considerations, including recruitment potential, funding constraints, and the high level of participant engagement required for an exercise intervention in this patient population. This decision was also informed by previous feasibility studies investigating exercise interventions in cancer populations with similar feasibility outcomes [45–48]. Recommendations for pilot trials typically suggest a sample size of 10 to 40 participants per group [49–51]. Our 2:1 randomization design (14 interventions, 7 controls) does not fully align with these recommendations due to practical constraints and the need to prioritize intervention feasibility while still maintaining a control group for study design experience and exploratory comparisons. However, the total sample size of 21 participants is consistent with feasibility study guidelines, which emphasize that smaller sample sizes are appropriate when assessing process-related outcomes rather than effect sizes [44].

In the interest of transparency, we acknowledge the precision limitations of our feasibility estimates. Assuming an 80% retention rate with *N* = 21, the 95% confidence interval ranges from 62 to 98%. This wide interval is an inherent limitation of feasibility studies but does not invalidate the study’s objectives. While retention rates in pilot trials are often used to inform sample size calculations for definitive trials, this is not the objective of our study. Instead, our focus is on determining whether trial procedures are practical and achievable within study context.

### Intervention

The intervention consists of a 12-week exercise program that features three weekly, 2-h training sessions at the Steno Diabetes Centre, Aarhus, facilitated by a physiotherapist experienced in working with cancer patients. The training sessions consist of a joint warm-up session and functional stability exercises (30 min), followed by individual cardiovascular and resistance training (1 h), ending with a joint stretching session (15 min). The cardiovascular training consists of short but high-intensity sessions, aiming for a Borg Rating of Perceived Exertion of 14–20 [52]. The duration of cardiovascular training is increased each week, combined with an increased number of high-intensity intervals, adjusted individually. Progressive resistance training is conducted as circuit training of three sets incorporating both weight machines and floor exercises. The load is gradually increased over time with decreasing repetitions: weeks 1–4 doing 12 repetitions, weeks 5–8 doing 10 repetitions, and weeks 9–12 doing 8 repetitions. The last 15 min of each training session are spent on socializing and evaluating the training. Participants will only be discontinued from the study in the case of the training being assessed as unsafe by study personnel with medical background.

### Control group

Participants assigned to the control group receive usual care at the Department of Oncology. They will undergo all study measurements at the defined timepoints, similar to the intervention group. For ethical reasons, the participants are informed of the possibility of being referred to training and rehabilitation at municipal service providers through the Department of Oncology. The participants will receive advice regarding training from the physiotherapist during baseline assessments.

### Study timeline

Recruitment of participants started in late February 2024. The project is scheduled to conclude at the latest in June 2025. The 24-week study period includes four assessment time points: baseline (T1), midpoint of the exercise program after 6 weeks (T2), completion of the exercise program at 12 weeks (T3), and a 12-week follow-up after the intervention period (T4).

### Baseline data

After obtaining written consent, baseline data are collected. Information regarding age, ECOG performance status, current medications, location of metastases, and contact information are registered in REDCap [42]. Furthermore, the participants are mailed an electronic baseline questionnaire including questions on demographic information (living arrangements, educational level, and employment status), health information (comorbidities and current medications), and lifestyle factors (current physical activity level, barriers to and facilitators of exercise, diet, and alcohol consumption). The questionnaire is based on the baseline questionnaire used in the Body & Cancer Program [53] and a dietary questionnaire used at Steno Diabetes Center Aarhus.

### Feasibility outcomes

Multiple feasibility outcomes are investigated in this study:

- Recruitment rate, consisting of the proportion of eligible participants who choose to participate in the study.
- Adherence to the exercise program, measured as the attendance rate.
- Retention, defined as the percentage of consenting participants in the intervention and control group completing T3.
- Acceptability, defined as the participants’ satisfaction with the study and intervention, employing both quantitative (ad hoc questions using Likert-scale scoring and open-ended questions) and qualitative (semi-structured interviews) methods.
- Adherence to other study procedures, i.e., the rate of completed questionnaires and collected biomarker samples.

### Effect outcomes

Multiple effect outcomes will be explored as described below.

#### Metabolic health

Metabolic health is estimated by calculating a metabolic score. The metabolic score consists of mid-level blood pressure [(systolic blood pressure + diastolic blood pressure)/2], glucose, and triglycerides. The score will be calculated as the sum of Z-transformed levels, with glucose and triglycerides log-transformed. This method of using metabolic score is also applied in Sun et al. [54] and Dieli-Conwright et al. [36]. Blood pressure is measured with an automated blood pressure cuff with the subjects seated quietly for at least 5 min before measuring three times, with 2 min between each measurement [55]. The average of the last two blood pressure recordings is used if the difference in systolic blood pressure is less than 5 mmHg.

#### Body composition

- Bioelectrical impedance analysis is measured with the TANITA MC-780MA-N multifrequency segmental body composition monitor to assess changes in weight, BMI, fat mass, muscle mass, visceral fat, basal metabolic rate, and total body water [56].
- Waist and hip circumference are measured according to the World Health Organization (WHO) [57], measuring waist circumference at the midpoint between the lower margin of the last palpable rib and the iliac crest and hip circumference around the widest portion of the buttocks. A waist-hip ratio is calculated.

#### Obesity-related biomarkers

Fasting blood samples (> 4 h of fasting) are assessed for levels of the following biomarkers: high-density lipoprotein, low-density lipoprotein, triglycerides, total cholesterol, plasma glucose, HbA1c, thyroid stimulating hormone, free T4, total T3, C-reactive protein, total leucocytes, differential count of leukocytes, albumin, alanine aminotransferase, hemoglobin, and thrombocytes.

#### Physical performance

- Hand grip strength (HGS) is measured with a Saehan DHD-1 digital hand dynamometer. HGS is correlated with all-cause mortality, overall functional status, and physical performance in patients with advanced cancer [58]. In this study, HGS is measured in both hands at least three times while the participant is in a seated position with the arm concerned laying on an armrest and the wrist in a neutral position [59].
- Maximal oxygen uptake (VO^2^_max_) and fitness rating are assessed with a Watt-max test on a cycle ergometer [60] performed by an experienced physiotherapist or trained personnel.
- Sit-to-stand in 30 s is measured following a test manual from the Danish Association of Physiotherapists [61]. It is a validated measure of physical performance and lower limb power [62, 63].

#### Patient-reported outcomes

- HRQoL is assessed with the cancer-specific European Organisation for Research and Treatment of Cancer (EORTC) QLQ-C30 (version 3) questionnaire [64]. The QLQ-C30 is composed of both multi-item scales and single-item measures. These include five functional scales, three symptom scales, a global health status/QoL scale, and six single items, with 30 items in total. All scales and single-item measures range in score from 0 to 100. A high score on the functional scale represents a high level of functioning, a high score for the global health status represents a high HRQoL, but a high score on a symptom scale represents a high level of symptomatology [65].
- Self-efficacy in coping with cancer is assessed with the 12-item Cancer Behaviour Inventory (CBI-12) (version 2.0) [66, 67]. The questionnaire is scored on a one-dimensional scale, summing up the ratings for the items. A higher score indicates higher levels of cancer-related self-efficacy, with a maximum score of 108.
- Sleep quality is assessed with the Pittsburgh Sleep Quality Index (PSQI) [68], which has been validated in cancer populations [69]. The questionnaire consists of 19 items grouped into seven component scores. The directionality and interpretation of scores vary across items. A total score ranging from 0 to 21 can be calculated, with a higher score indicating poorer sleep quality. A score < 5 indicates good sleep quality.
- Insomnia is assessed with the Insomnia Severity Index (ISI) [70], which has also been validated in cancer patients [71]. It contains 7 items and is scored on a one-dimensional scale with a maximum score of 28. The total score is grouped into four categories: no insomnia (0–7), subclinical insomnia (8–14), clinical insomnia (15–21), and severe clinical insomnia (22–28).
- Sleep patterns, including sleep onset latency (SOL), wake after sleep onset (WASO), early morning awakenings (EMA), time in bed (TiB), total sleep (TST), and sleep efficiency (TST/TiB*100) are measured using the Consensus Sleep Diary [72] 5 days in a row at the three time-points (T1, T2, and T3).

### Adverse events

Adverse events related to the study procedures will be continuously collected throughout the study. In the case of a serious adverse event, this will be reported within a day to study sponsor. Assessment, grading, and reporting of adverse events will follow the detailed guideline from the Exercise Harms Reporting Method (ExHaRM) [73], developed specifically for exercise interventions in oncology populations.

#### Actigraphy

Daily physical activity and circadian activity rhythms are evaluated with objective measures of rest/wake activity cycles recorded with the ActTrust AT0503 Wrist Actimeter. As we currently have access to only two actigraphs, a randomly selected subset of patients in the intervention arm are asked to continuously wear the actigraph on their non-dominant wrist for a duration of 12 weeks to gain experience using actigraphy tools for data collection in this study design.

### Progression criteria

To ensure that the feasibility objectives in this study are met, progression criteria for each objective are developed [74]. Defining progression criteria is necessary in the interpretation of trial findings. For this purpose, a traffic light system is employed. In this system, a lower (red) threshold indicates major problems that require urgent attention (and perhaps are unfixable), amber indicates minor problems that require attention, and green indicates areas of no concern [74]. As estimates of rates in the feasibility measures are subject to uncertainty, this system allows for chance variation in contrast to definitive thresholds. We will identify fundamental feasibility measures to accurately weigh the importance of each outcome in the assessment. In Table 1, the progression criteria for each outcome are outlined. These criteria are based on relevant studies, as referenced in the background section, and informed by prior research on feasibility outcomes in exercise interventions for breast cancer populations. To provide an overall assessment of the feasibility of the study, we employ a holistic approach, using quantitative measurements of the progression criteria, data from the evaluation questionnaire, and data on feasibility from the qualitative interviews collectively.

**Table 1:** Progression criteria for feasibility objectives

Feasibility objective	Red Major problem	Amber Minor problem	Green No concern	Fundamental objective
Recruitment rate	< 25%	25–33%	≥ 33%	Yes
Attendance rate	< 70%	70–80%	≥ 80%	Yes
Retention rate	< 65%	65–80%	≥ 80%	Yes
Acceptability of intervention^a^	< 75%	75–90%	≥ 90%	Yes
Adherence to other study procedures	< 80%	80–90%	≥ 90%	No

Lower and upper threshold values in the traffic light system for feasibility outcomes

^a^Percentage of participants in the intervention group that answers “Yes” to the question: “Would you recommend others to participate in the physical activity program?” in the evaluation questionnaire

#### Participant evaluation surveys

Two questionnaires are used to assess study satisfaction—one for the intervention group and one for the control group. The questionnaire for the intervention group consists of 28 items and investigates different feasibility dimensions: (1) acceptability in terms of satisfaction with the training itself, the exercise equipment, group training, and the intervention overall, and (2) feasibility of the study design, including the amount and length of training sessions, the length of the exercise program and the feasibility of other study procedures. The evaluation questionnaire in the control group is shorter, with only seven items, and investigates acceptability in terms of being randomized to the control group and the load of study procedures.

#### Qualitative evaluation

All participants in the intervention arm are invited to participate in semi-structured qualitative interviews with a clinical nurse specialist (PS). An interview guide is constructed for this purpose, drawing inspiration from relevant studies in the construction hereof [75–77]. Four main areas are investigated: (1) motivation, facilitation, and exercise preferences; (2) barriers to exercise, (3) participation in the intervention, and 4) exercise in the future. These four main areas each investigate multiple aspects of exercise interventions, both generally and in our study. Specific questions in the interview guide are aimed at evaluating feasibility, particularly acceptability. In Table 2, examples of questions from the interview guide that explores feasibility are presented alongside the related evaluation criteria.

**Table 2:** Interview questions concerning feasibility outcomes and the respective evaluation criteria

Question	Feasibility dimension	Evaluation criteria
**Did the intervention motivate you?**	Acceptability	If the majority of the participants gained motivation from the intervention
**Have you experience**d** personal challenges by participating in the intervention?**	Acceptability	None or few preventable personal challenges due to participation
**Did the study meet your expectations?**	Acceptability	If the study met or exceeded the expectations in majority of the participants
**Is there anything we could do differently?**	Overall feasibility	No suggestion of major changes
**What could you imagine would be beneficial for us to do to include women in your situation in our study?**	Study design	No suggestion of major changes

### Statistical analysis plan

Data are entered into a REDCap database in real time using electronic case report forms.

Baseline data on participant demographics and characteristics will be presented in a descriptive table in accordance with the CONSORT guidelines [43]. For feasibility outcomes, the analysis will be mainly descriptive and focus on confidence interval estimation and not formal hypothesis testing, in accordance with the CONSORT extension to randomized feasibility and pilot trials [40].

The results of the effect measurements will be presented as means, standard deviations, and effect sizes (standardized mean differences) to assess potential effects, but no formal statistical tests will be performed. Effects will be analyzed at T3 (post-intervention), and further analysis will be made to see if these effects sustain until T4 (12-week follow-up post intervention). The semi-constructed interviews will be digitally recorded, transcribed, and analyzed using thematic analysis [78]. Statistical analyses will be carried out using the Stata 18 software [79].

## Discussion

Patients with MBC often experience a decrease in HRQoL and reduced physical functioning, and patients with ER + disease receiving endocrine-based treatment also have an increased risk of weight gain and adverse metabolic effects. To reduce cancer- and treatment-related side effects, exercise is recommended for BC patients in the curative and adjuvant settings. In contrast, evidence in the metastatic setting remains limited, and no study has yet investigated exercise in endocrine-treated patients with MBC and overweight. The overall purpose of the present study is to develop a feasible exercise intervention to support improved metabolic health, body composition, and patient-reported outcomes for MBC patients in endocrine therapy with overweight.

The intervention design is based on evidence from the curative and adjuvant BC settings. Exercise programs lasting 12 weeks or longer are reported to have positive effects by using combined aerobic and resistance training [80]. Furthermore, our intervention is designed to be intense, but at the same time not too time-consuming, deciding on three weekly training sessions for 12 weeks. The training sessions are planned to take place in the afternoon on weekdays to accommodate patients with MBC who still work [81].

We opted for an unequal randomization ratio (2:1) to enhance the assessment of feasibility objectives, such as adherence, retention, and acceptability, which are directly tied to the intervention. This design provides greater precision in evaluating these outcomes compared to a 1:1 ratio. Additionally, the unequal allocation addresses ethical considerations by minimizing the number of participants assigned to the control group, ensuring more participants benefit from the intervention. The control group remains essential for collecting data on comparator group design, bias reduction, and addressing ethical dilemmas for a future trial.

Existing literature on feasibility trials is generally inconsistent in terms of how to design appropriate outcomes and assess overall feasibility. The feasibility outcomes in this study were chosen on the basis of other feasibility trials and relevant guidelines. To quantify feasibility outcomes, we designed progression criteria for each outcome. To achieve a more nuanced feasibility assessment, we also include qualitative interviews, which will help us gain deeper insight into motivators, preferences, facilitators, and barriers for exercise in patients with MBC and overweight to better understand their needs, which might differ from early-stage BC patients.

The evaluation of overall feasibility will include all study procedures, including evaluating the suitability of screening and inclusion procedures, inclusion and exclusion criteria, study objectives, and the acceptability of the intervention and measurements performed. We will use the gained knowledge on feasibility and acceptability to improve the final study protocol and assess whether to move forward with the full-scale study. This assessment will be made by the steering committee including an oncologist, psychologist, oncology nurse, and physiotherapist.

### Strengths and limitations

A methodological strength of the present feasibility study is that we follow good practice recommendations for pilot and feasibility studies [82] as well as the SPIRIT checklist for reporting the study protocol. Another strength is that we employ both self-reported and objective data measurements to estimate effects, and we use quantitative and qualitative approaches to evaluate feasibility.

In the study design, we employ a randomized controlled study design, which is important for testing the feasibility of the overall study methodology and intervention. However, as the control group is small, with seven participants, the purpose hereof is to test the feasibility of including a usual care control group rather than conducting hypothesis testing. Another strength in the study design is the long period of follow-up and the use of multiple measurement points. Furthermore, we take precautionary measures to prevent missing data, such as sending reminder emails and providing physical reminders to participants. A strength of our exercise intervention is that it is delivered in person by highly skilled personnel experienced in working with cancer patients.

Some limitations should also be noted. First, with a sample size of 21 participants, the study is rather small and not powered for hypothesis testing. However, as a feasibility trial, the primary aim is not to detect statistical differences but to assess the practicality of conducting a larger trial. The chosen sample size was based on pragmatic considerations, including recruitment feasibility within the given timeframe, resource availability, and the high level of participant engagement required for an exercise intervention. While we recognize that the precision of feasibility estimates is inherently limited in small feasibility studies, our approach incorporates both quantitative and qualitative data that we are confident will provide a comprehensive evaluation.

Second, owing to the study design and intervention, blinding of participants and study personnel is not possible during study enrollment and participation. This could make the study susceptible to performance bias. However, we have employed multiple objective outcome measures, which reduce the influence of performance bias. To minimize performance bias in study personnel, the same procedure and guidelines when performing measurements are followed each time despite group allocation of the participants. Selection bias is likewise a concern, as we expect patients to be less likely to participate if they live further away from the hospital, have a full-time job, have limited transportation possibilities, or are older. Therefore, the included participants may not be representative of all endocrine-treated patients with MBC and overweight.

Finally, most exercise intervention studies are at risk of contamination in the control group, as participants randomized to the control group may increase their amount of exercise. This is a concern of the current study. It is suggested that in studies where the control group is offered an intervention rather than usual care only during or after an intervention period, the contamination rate is greatly reduced e.g. by providing accelerometers in the control arm [83]. In this feasibility study, the control group is not offered any intervention; however, we will test the use of accelerometers to possibly implement in a full-scale study in both study arms and thereby decrease the risk of contamination.

In conclusion, this study is an important first step to gain novel insights into the feasibility of exercise in endocrine-treated patients with MBC and overweight. The results from this protocol will decide and guide a future definitive randomized controlled study.

### Trial status

Study recruitment began on February 19, 2024. As of now, 19 out of 21 participants have been included.

### Dissemination

The results of the study will be published in an international peer-reviewed scientific journal, presented at relevant national and international conferences, presented at patient associations, and disseminated in social media outlets. The results will be published regardless of being positive, negative, or inconclusive.

### Modification of the protocol

Any modifications to the protocol which may impact on the conduct of the study, potential benefit of the patient or may affect patient safety will require a formal amendment to the protocol. Such amendment will be agreed upon in the study group and approved by the Central Denmark Region Ethics Committee.

### Trial sponsor

Name: Aarhus University Hospital.

Address: Palle Juul-Jensens Boulevard 99, DK-8200, Aarhus N, Denmark.

Telephone: + 45 7845 000.

Email: auhhov@rm.dk.

## Supplementary Information

Additional file 1: SPIRIT Outcomes 2022 Checklist: Recommended items to address in a clinical trial protocol and related documents.

## Data Availability

Not applicable as this current manuscript does not contain any data or materials.

## Abbreviations

AIs: Aromatase inhibitors
BC: Breast cancer
BMI: Body‒mass index
CBI: Cancer Behaviour Index
CONSORT: Consolidated Standards of Reporting Trials
EMA: Early morning awakenings
ECOG: Eastern Cooperative Oncology Group
EORTC: European Organisation for Research and Treatment of Cancer
ER +: Estrogen receptor positive
HRQoL: Health-related quality of life
ISI: Insomnia Severity Index
MBC: Metastatic breast cancer
SOL: Sleep onset latency
PSQI: Pittsburgh Sleep Quality Index
REDCap: Research Electronic Data Capture
SPIRIT: Standard Protocol Items: Recommendations for Interventional Trials
TiB: Time in bed
TST: Total sleep
WASO: Wake after sleep onset
WHO: World Health Organization
